# Childhood adversity, mental health and suicide (CHASE): a longitudinal case-control linked data study of lifetime inpatient records associated with suicide.

**DOI:** 10.23889/ijpds.v7i3.1878

**Published:** 2022-08-25

**Authors:** Jan Savinc, Nadine Dougall, Margaret Maxwell, Thanos Karatzias, Rory O'Connor, Brian Williams, Ann John, Helen Cheyne, Claire Fyvie, Jonathan Bisson, Carina Hibberd, Susan Abbott-Smith, Liz Nolan

**Affiliations:** 1 Edinburgh Napier University; 2 University of Stirling; 3 University of Glasgow; 4 University of the Highlands & Islands; 5 Swansea University; 6 The Rivers Centre, NHS Lothian; 7 Cardiff University; 8 Child and Adolescent Mental Health Service (CAMHS), NHS Lothian; 9 Aberlour

## Objectives

Childhood adversity (CA) carries an increased risk of developing later mental health (MH) problems and suicidal behaviour. This study aimed to summarise lifetime hospital attendances for CA and MH in young people who later died by suicide.

## Approach

This study is a retrospective longitudinal case control study. Lifetime Scottish inpatient acute and psychiatric records were linked to death records and summarised for individuals born since 1981 who died by suicide in the period 1991-2017 (cases), and controls (1:10) matched on sex, age, and postcode. Relevant records were coded MH (including self-harm) and/or CA. Descriptive statistics and odds ratios (OR) were computed.

## Results

Data for 2,477 and 24,777 ‘cases’ and ‘controls’ were extracted, of whom 2,106 cases (85%) and 13,589 controls (55%) had lifespan hospital records. Mean age at death for cases was 23.7 (SD=4.9) and 75.9% were male. Psychiatric records represented 11.6% and 1.4% of records for cases and controls, respectively.

For the age range 10-18, Maltreatment & violence-related codes were recorded for 160 (7.6%) cases and 371 (2.7%) controls, corresponding to OR=2.9 (95%CI: 2.4-3.6). This was compared with MH at 458 (21.7%) cases and 560 (4.1%) controls and OR=6.5 (95%CI: 5.7-7.4). The highest adjusted ORs were for self-harm episodes recorded in general hospital with aORmale=6.56 (95%CI: 4.96-8.68) and aORfemale=6.87 (95%CI: 4.99-9.48).

## Conclusion

All CA and MH presentations in inpatient hospital records were associated with greater risk of subsequent suicide, with the strongest association for self-harm.

